# Mental health conditions and risk of first‐ever ischaemic stroke and death in patients with incident atrial fibrillation: A nationwide cohort study

**DOI:** 10.1111/eci.13801

**Published:** 2022-05-07

**Authors:** Konsta Teppo, Jussi Jaakkola, Fausto Biancari, Olli Halminen, Jukka Putaala, Pirjo Mustonen, Jari Haukka, Miika Linna, Janne Kinnunen, Paula Tiili, Elis Kouki, Tero Penttilä, Juha Hartikainen, Aapo L. Aro, K. E. Juhani Airaksinen, Mika Lehto

**Affiliations:** ^1^ University of Turku Turku Finland; ^2^ Heart Unit Satakunta Central Hospital Pori Finland; ^3^ Heart and Lung Center Helsinki University Hospital Helsinki Finland; ^4^ Clinica Montevergine GVM Care & Research Mercogliano Italy; ^5^ Department of Industrial Engineering and Management Aalto University Espoo Finland; ^6^ Neurology Helsinki University Hospital, and University of Helsinki Helsinki Finland; ^7^ Heart Center Turku University Hospital Turku Finland; ^8^ University of Helsinki Helsinki Finland; ^9^ Aalto University Espoo Finland; ^10^ University of Eastern Finland Kuopio Finland; ^11^ Heart Hospital, Tampere University Hospital Tampere Finland; ^12^ Heart Center Kuopio University Hospital Kuopio Finland; ^13^ Lohja Hospital Department of Internal Medicine Lohja Finland

**Keywords:** atrial fibrillation, ischaemic stroke, mental health conditions, mortality, psychiatric disorders

## Abstract

**Background:**

Atrial fibrillation (AF) patients with mental health conditions (MHCs) have higher incidence of ischaemic stroke (IS) than patients without MHC, but whether this results from direct impact of MHCs or relates to higher prevalence of comorbidities and differences in the use of oral anticoagulant (OAC) therapy is unclear. We assessed the hypothesis that MHCs independently increase the risk of IS in patients with incident AF.

**Methods:**

The nationwide FinACAF cohort covered all 203,154 patients diagnosed with incident AF without previous IS or transient ischaemic attack in Finland during 2007–2018. MHCs of interest were depression, bipolar disorder, anxiety disorder, schizophrenia and any MHC. The outcomes were first‐ever IS and all‐cause death.

**Results:**

The patients' (mean age 73.0 ± 13.5 years, 49.0% female) mean follow‐up time was 4.3 (SD 3.3) years and 16,272 (8.0%) experienced first‐ever IS and 63,420 (31.2%) died during follow‐up. After propensity score matching and adjusting for OAC use, no MHC group was associated with increased IS risk (adjusted SHRs (95% CI): depression 0.961 (0.857–1.077), bipolar disorder 1.398 (0.947–2.006), anxiety disorder 0.878 (0.718–1.034), schizophrenia 0.803 (0.594–1.085) and any MHC 1.033 (0.985–1.085)). Lower rate of OAC use partly explained the observed higher crude IS incidence in patients with any MHC. Depression, schizophrenia and any MHC were associated with higher all‐cause mortality (adjusted HRs [95% CI]: 1.208 [1.136–1.283], 1.543 [1.352–1.761] and 1.149 [1.116–1.175], respectively).

**Conclusions:**

In this nationwide retrospective cohort study, MHCs were not associated with the incidence of first‐ever IS in patients with AF.

## 1 INTRODUCTION

Atrial fibrillation (AF) is the most common sustained arrhythmia with a prevalence as high as 4.1%, and the prevalence is estimated to further increase substantially owing to improved life expectancy and advances in AF diagnostics.^1^, ^2^, ^3^ AF is the leading cause of ischaemic stroke (IS) and is associated with increased mortality.^4^ Fortunately, in AF patients with stroke risk factors, oral anticoagulant (OAC) therapy can effectively reduce the risk of IS and death.^5^ The well‐validated CHA_2_DS_2_‐VASc score is used to identify patients at high risk of stroke and indicated to receive OACs for stroke prevention; however, there are several other stroke risk factors that are not included in this score, such as impaired renal function, malignancies and dyslipidaemia.^6^ Interestingly, mental health conditions (MHCs), particularly depression, bipolar disorder, anxiety disorder and schizophrenia, have been shown to increase IS risk, regardless of the presence of AF.^7^, ^8^, ^9^, ^10^ However, in patients with AF, the evidence on the impact of these MHCs on the incidence of IS and mortality is limited and has shown inconsistent results.^11^, ^12^, ^13^ Additionally, AF patients with MHCs are less likely to receive OAC therapy for stroke prevention than patients without MHC, but the impact of this poorer OAC coverage on outcomes is unknown.^14^ The objective of the present nationwide cohort study was to assess the incidence of first‐ever IS in patients with and without MHCs and the independent effect of different MHCs on IS risk in patients with incident AF. Additionally, we aimed to assess all‐cause mortality rates in AF patients with and without MHCs and whether differences in OAC use affect outcomes in these patients.

## 2 METHODS

### 2.1 Study population

The Finnish AntiCoagulation in Atrial Fibrillation Study (FinACAF) (ClinicalTrials Identifier: NCT04645537; ENCePP Identifier: EUPAS29845) is a retrospective nationwide registry‐based cohort study covering all patients with diagnosed AF in Finland during 2004–2018.^1^ Patients were identified from three national health care registers (hospitalizations and outpatient specialist visits: HILMO; primary health care: AvoHILMO; and National Reimbursement Register upheld by Social Insurance Institute: KELA). The inclusion criterion for the cohort was an International Classification of Diseases, Tenth Revision (ICD‐10) diagnosis code I48 (including atrial fibrillation and atrial flutter, together referred as AF) recorded between 2004 and 2018 in any of the named registers and cohort entry occurred on the date of the first recorded AF diagnosis. Follow‐up continued until death or 31 December 2018, whichever occurred first. The exclusion criteria were age < 18 years at AF diagnosis and permanent migration abroad before 31 December 2018. The present substudy focussed on patients with incident AF during 2007–2018 without history of IS or transient ischaemic attack (TIA) at the time of cohort entry. The study was conducted within a cohort of patients with incident AF, established in a previous study within the FinACAF cohort.^14^ The patient selection process is presented in Figure S1.

### 2.2 Mental health conditions

Mental health conditions (MHCs) of interest were depression, bipolar disorder, anxiety disorder and schizophrenia. These specific diagnoses were chosen due to their high prevalence and burden in the ageing population of patients with AF.^15^ Patients were classified into diagnostic groups if they were recorded with the ICD‐10 diagnosis code or International Classification of Primary Care, Second Edition (ICPC‐2) entry of the condition before the index date as follows: depression (ICD‐10: F32, F33, F34.1; ICPC‐2: P76), anxiety disorder (ICD‐10: F40‐F42, F43.1; ICPC‐2: P74), bipolar disorder (ICD‐10: F31; ICPC‐2: P73) and schizophrenia (ICD‐10: F20; ICPC‐2: P72). Patients with more than one of these conditions were classified into each diagnostic category separately. Patients were classified to have any MHC if they had any of these four MHCs, and additionally, due to the possible information bias from inaccurate recording of MHC diagnoses, patients who had fulfilled a prescription of an antidepressant, antipsychotic or mood‐stabilizing medication within the year before the index date were classified to have any MHC (Anatomical Therapeutic Chemical codes: N05A, N05BE01, N06A). Medication data were not utilized to further classify patients into specific conditions.

### 2.3 Outcomes

The primary outcome, first‐ever IS, was considered to occur on the first date of a recorded I63 ICD‐10 diagnosis code after cohort entry. The codes and dates were searched from the aforementioned hospital and primary care registers, as well as from the National Death Register upheld by Statistics Finland. The presence and dates of the secondary outcome, all‐cause death, were obtained from the National Death Register.

### 2.4 Study ethics

The FinACAF study was approved by the Ethics Committee of the Medical Faculty of Helsinki University, Helsinki, Finland (nr. 15/2017) and granted research permission from the Helsinki University Hospital (HUS/46/2018). Respective permissions were obtained from the Finnish register holders (KELA 138/522/2018; THL 2101/5.05.00/2018; Population Register Centre VRK/1291/2019–3; Statistics Finland [TK‐53‐1713‐18 / u1281]; Tax Register VH/874/07.01.03/2019)). The patients' identification numbers were pseudonymized, and informed consent was waived due to the retrospective registry nature of the study. The study conforms to the Declaration of Helsinki as revised in 2002 and reporting of the study conforms to broad EQUATOR guidelines.^16^

### 2.5 Statistical analysis

Statistical analyses were performed with the IBM SPSS Statistics software (version 27.0, SPSS, Inc.), Stata v. 15.1 (StataCorp LLC) and R (version 4.0.5, https://www.R‐project.org). The chi‐square test was used to compare differences between proportions and the independent samples t‐test to analyse continuous variables. Poisson regression was used to determine crude incidence and incidence rate ratios (IRRs) of IS as well as crude mortality and mortality rate ratios (MRRs) separately for each MHC category.

Since an imbalance between the study cohorts was observed in the overall series, propensity score matching was performed to obtain study cohorts balanced for baseline variables. Propensity scores were calculated separately for each MHC category using a non‐parsimonious logistic regression with MHCs as dependent variables including the following variables into the regression models: age, gender, calendar year of AF diagnosis, hypertension, dyslipidaemia, heart failure, diabetes, vascular disease, renal failure or dialysis, liver cirrhosis or failure, alcohol abuse, income (highest annual income during follow‐up divided in quintiles), CHA_2_DS_2_‐VASc score and modified HAS‐BLED score (without points from labile INR or concomitant antiplatelet/NSAIDs use). The definitions of the comorbidities are presented in Table S1. One‐to‐one matching was performed with the nearest neighbour approach using a calliper width of 0.1, that is, 0.2 of the standard deviation of the logit (0.55). Standardized difference <0.10 was considered an acceptable balance between the study cohorts.

Observation of an IS event might be hindered by mortality occurring during the study period, and therefore, a Fine‐Grey regression model with all‐cause death as competing event was used to estimate subdistribution hazard ratios (SHRs) of IS in the propensity score matched pairs for each MHC category and additionally for patients with any MHC in the overall series. Similarly, hazard ratios (HRs) of all‐cause mortality were calculated using the Cox proportional hazards regression in the matched pairs for each MHC category, as well as for patients with any MHC in the overall series. To assess the effect of OAC therapy on outcome events, the Fine‐Grey and Cox regression models were thereafter further adjusted with initiation of OAC therapy during follow‐up and before possible IS.

## 3 RESULTS

Overall, 203,154 patients (49.0% female) with incident AF without history of stroke or TIA were identified. The mean age of the patients was 71.8 years (SD 13.5), and the overall prevalence of any MHC at cohort entry was 19.2% (38,942 patients). Patients with MHCs were more often female and had lower income and higher prevalence of cardiovascular risk factors than patients with no history of MHC (Table 1). The mean follow‐up time was 4.3 (SD 3.3) years, and 16,272 (8.0%) patients experienced their first‐ever IS and 63,420 (31.2%) patients died during follow‐up. The primary cause of death was cardiovascular disease in 31,323 patients (ICD‐10 codes I00‐I99; 49.4% of deaths) and cancer in 12,369 patients (ICD‐10 codes C00‐I99 and D00‐D48; 19.5% of deaths). The first recording of IS was obtained from the hospital register in 13,715 (84.3%) patients, from the primary care register in 1675 (10.3%) patients and from the causes of death in 882 (5.4%) patients. During follow‐up, OAC therapy was initiated less often in patients with any MHC than in patients without (63.8% vs. 72.1%, p < 0.001, Table 1, Tables S1 and S3). Propensity score matching for each MHC category resulted in pairs with similar characteristics as demonstrated by standardized differences less than 0.10 in all used covariates (Tables S1 and S3).

**TABLE 1:** Descriptive characteristics of the cohort before propensity score matching according to the presence of MHCs

	No MHC	Any MHC	Depression	Bipolar disorder	Anxiety disorder	Schizophrenia
	*n* = 164,212	*n* = 38,942	*n* = 8982	*n* = 933	*n* = 3689	*n* = 1329
Demographics						
Mean age, years	71.8 (13.3)	71.7 (14.6)	68.7 (14.6)*	62.8 (12.8)*	64.8 (16.6)*	68.8 (11.7)*
Female sex	76,534 (46.6)	22,962 (59.0)*	5265 (58.6)*	412 (44.2)	2221 (60.2)*	636 (47.9)
Income quintiles		*	*	*	*	*
1st	31,183 (19.0)	9852 (25.3)	1859 (20.7)	187 (20.0)	714 (19.4)	712 (53.6)
2nd	30,892 (18.8)	8710 (22.4)	2077 (23.1)	224 (24.0)	871 (23.6)	350 (26.3)
3rd	32,047 (19.5)	7905 (20.3)	2021 (22.5)	194 (20.8)	822 (22.3)	162 (12.2)
4th	34,410 (21.0)	6979 (17.9)	1819 (20.3)	185 (19.8)	781 (21.2)	61 (4.6)
5th	35,680 (21.7)	5496 (14.4)	1206 (13.4)	143 (15.3)	501 (13.6)	44 (3.3)
Comorbidities and medications						
Alcohol abuse	4344 (2.6)	3724 (9.6)*	1601 (17.8)*	271 (29.0)*	650 (17.6)*	129 (9.7)*
Diabetes	33,314 (20.3)	9322 (23.9)*	2389 (26.6)*	293 (31.4)*	811 (22.0)*	466 (35.1)*
Dyslipidaemia	73,535 (44.8)	17,918 (46.0)*	4274 (47.6)*	440 (47.2)	1623 (44.0)	464 (43.9)*
Heart failure	27,113 (16.5)	7780 (20.0)*	1633 (18.2)*	153 (16.4)	564 (15.3)*	412 (31.0)*
Hypertension	124,677 (75.9)	31,097 (79.9)*	7239 (80.6)*	725 (77.7)	2955 (80.1)*	890 (67.0)*
Liver cirrhosis or failure	789 (0.5)	321 (0.8)*	114 (1.3)*	11 (1.2)*	39 (1.1)*	10 (0.8)
Renal failure or dialysis	3046 (1.9)	906 (2.3)*	246 (2.7)*	22 (2.4)	90 (2.4)*	28 (2.1)
Vascular disease	39,071 (23.8)	10,111 (26.0)*	2249 (25.0)*	179 (19.2)	803 (21.8)	265 (19.9)*
CHA_2_DS_2_‐VASc score	3.0 (1.6)	3.3 (1.6)*	3.1 (1.7)*	2.5 (1.5)*	2.9 (1.7)*	3.1 (1.6)
Modified HAS‐BLED score(max 7)	1.7 (0.8)	1.8 (0.9)*	1.8 (0.9)*	1.7 (0.9)*	1.7 (0.9)*	1.6 (0.9)*
OAC therapy during follow‐up (before stroke)	118,111 (72.1)	24,781 (63.8)*	5760 (64.3)*	593 (63.8)*	2201 (59.9)*	788 (59.4)*

*Note:* Values denote n (%) or mean (standard deviation). Modified HAS‐BLED score, hypertension, abnormal renal or liver function, prior stroke, bleeding history, age > 65 years, alcohol abuse (no labile INR or concomitant antiplatelet/NSAIDs use, max score 7).

Abbreviations: CHA_2_DS_2_‐VASc, congestive heart failure, hypertension, age ≥ 75 years, diabetes, history of stroke or TIA, vascular disease, age 65–74 years, sex category (female); DOAC, direct oral anticoagulant; MHC, mental health condition; VKA, vitamin K antagonist.

*p < 0.05 when compared to patients without MHC.

**FIGURE 1:**
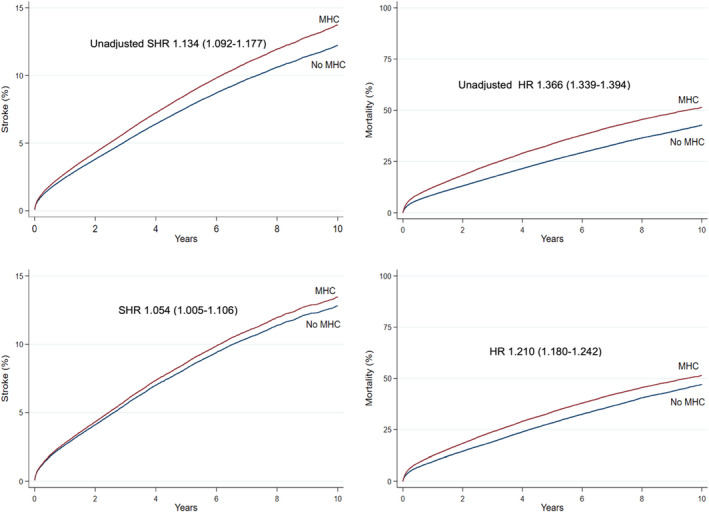
Cumulative incidence curves of first‐ever IS (left) and all‐cause death (right) before (upper panel) and after (lower panel) propensity score matching in patients with and without any MHC

**FIGURE 2:**
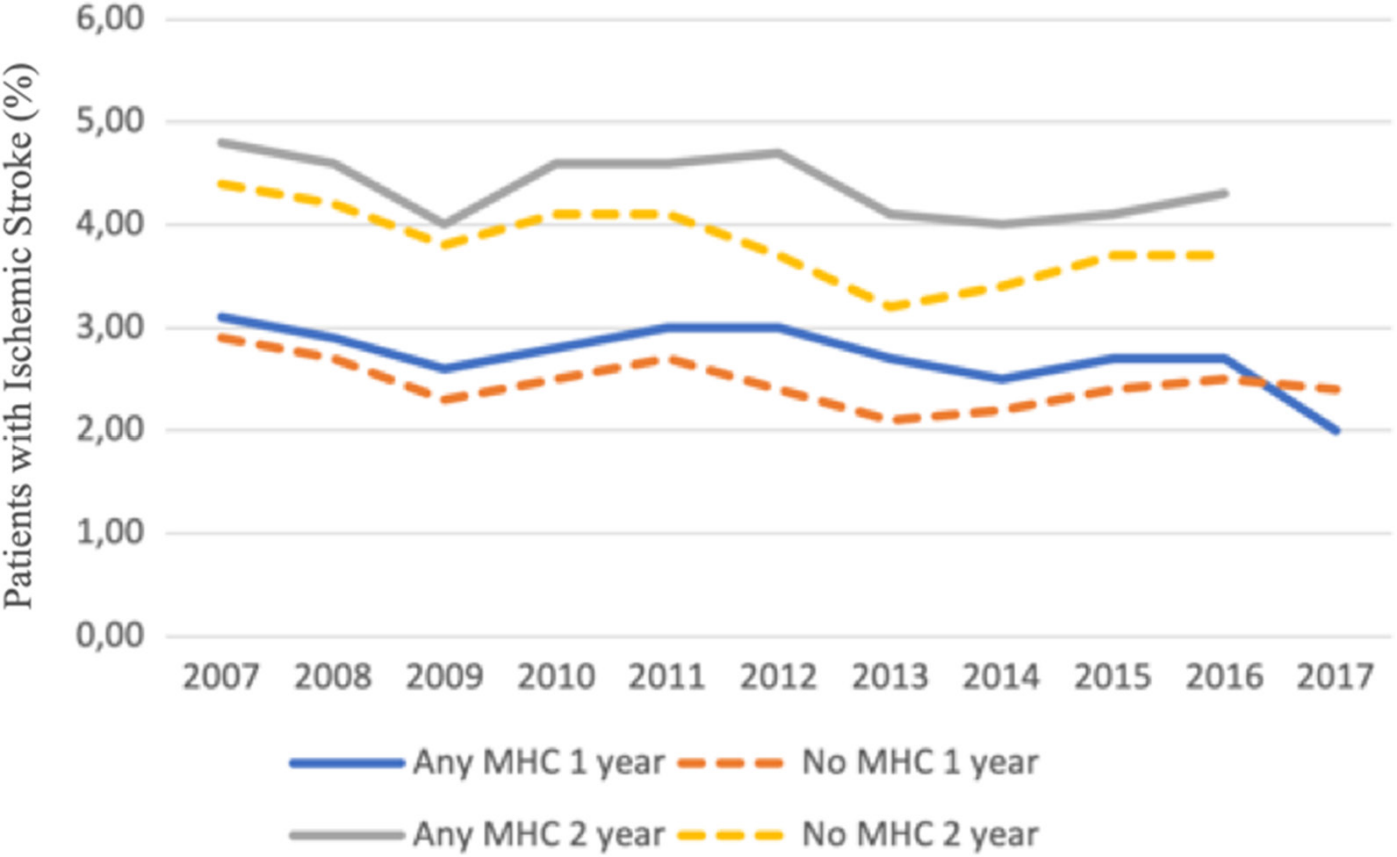
Proportions of patients experiencing first‐ever IS within one‐ and two‐year follow‐up according to the year of AF diagnosis in patients with and without any MHC

**FIGURE 3:**
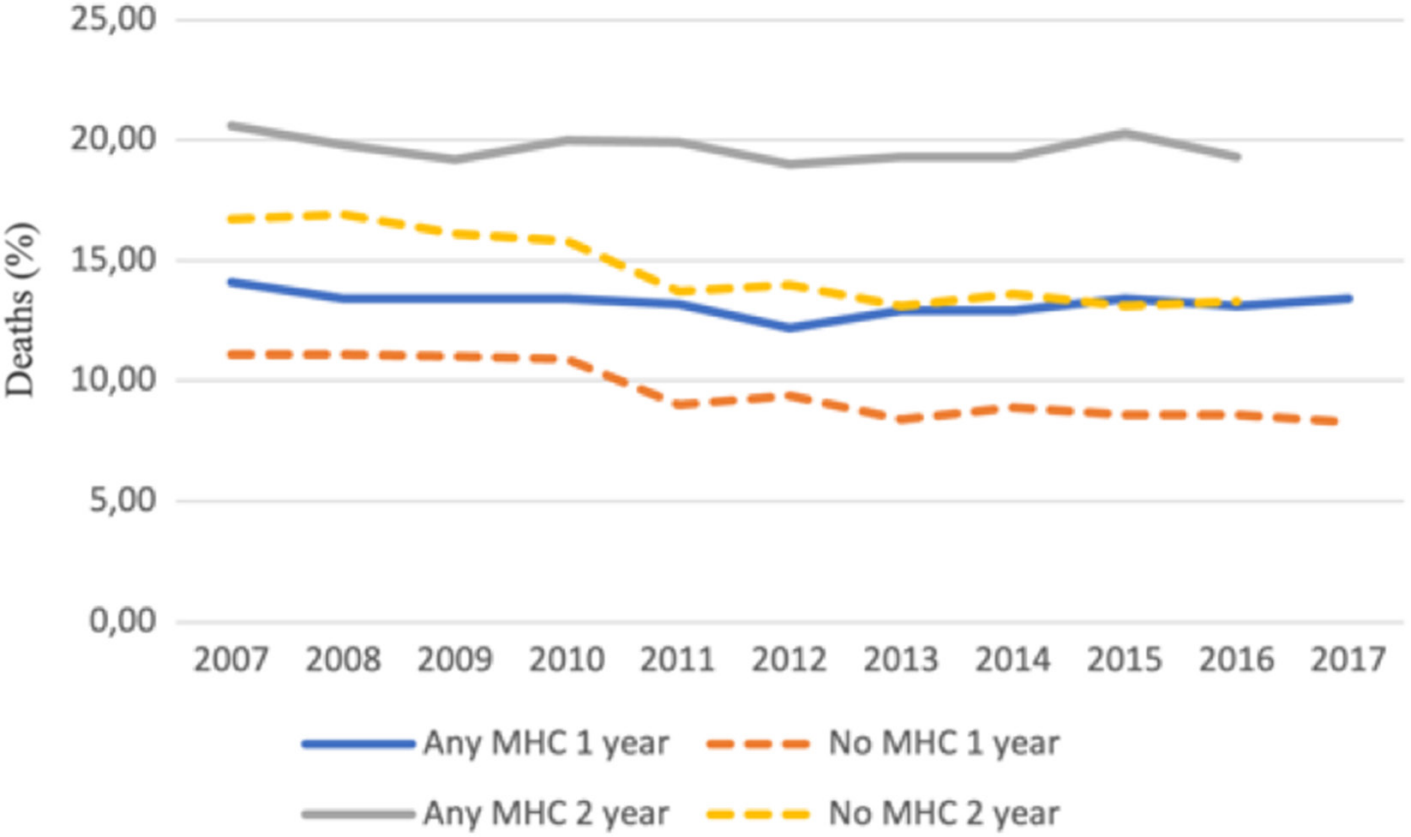
Proportions of patients experiencing death within one‐ and two‐year follow‐up according to the year of AF diagnosis in patients with and without any MHC

In the overall cohort before propensity score matching, the crude incidence of IS was higher in patients with any MHC but lower in patients with anxiety disorder, when compared to patients without MHCs. The crude IS incidence in patients with depression, bipolar disorder and schizophrenia did not differ significantly from patients without MHC (Figure 1, Table 2). On the contrary, the crude mortality was higher in patients with any MHC, depression or schizophrenia and lower in patients with anxiety disorder, while no difference was observed in patients with bipolar disorder, when compared to patients without MHC (Figure 1, Table 2). Both the crude IS incidence and mortality within 1‐ and 2‐year follow‐up since AF diagnosis were higher across the observation period in patients with any MHC than in those without MHC, except for the higher 1‐year stroke rate in patients without MHC in patients with AF diagnosed in 2017 (Figures 2 and 3).

**TABLE 2:** Crude incidence of IS and all‐cause mortality in the overall cohort before propensity score matching according to the presence of MHCs

Clinical condition	ISs (*n*)	Deaths (*n*)	Proportion of patients with IS	Proportion of died patients	P‐years until IS	P‐years until death	Incidence of IS (per 1000 p‐years)	Mortality (per 1000 p‐years)	Unadjusted IRR of IS	Unadjusted MRR
No MHC	12,810	48,773	7.8%	29.7%	691,456	718,613	18.5 (18.2–18.9)	67.9 (67.3–68.5)	(Reference)	(Reference)
Any MHC	3462	14,647	8.9%	37.6%	151,846	159,190	22.8 (22.1–23.6)	92.0 (90.5–93.5)	1.23 (1.19–1.28)	1.36 (1.33–1.38)
Depression	584	2578	6.5%	28.7%	30,060	31,200	19.4 (17.9–21.1)	82.6 (79.5–85.9)	1.05 (0.97–1.14)	1.22 (1.17–1.27)
Bipolar disorder	62	219	6.6%	23.5%	3396	3563	18.3 (14.2–23.4)	61.5 (53.8–70.2)	0.99 (0.77–1.27)	0.91 (0.79–1.03)
Anxiety disorder	184	756	5.0%	20.5%	12,147	12,496	15.1 (13.1–17.5)	60.5 (56.3–65.0)	0.82 (0.71–0.95)	0.89 (0.83–0.96)
Schizophrenia	90	612	6.8%	46.0%	4054	4171	22.2 (18.1–27.3)	146.7 (135.5–158.8)	1.20 (0.97–1.47)	2.16 (2.00–2.34)

*Note:* 95% confidence intervals in parenthesis. IRRs and MRRs estimated by Poisson regression. 95% confidence intervals in parenthesis.

Abbreviations: IRR, incidence rate ratio; IS, ischaemic stroke; MHC, mental health condition; MRR, mortality rate ratio; p‐year, patient year.

Among the propensity score matched pairs, none of the studied MHC groups were associated with the risk of first‐ever IS after adjusting for OAC use. Before adjusting for OAC use, any MHC was associated with a higher IS incidence; however, when OAC use was fitted in the competing risk model the risk estimate of any MHC for IS emerged nonsignificant (Figure 1, Table 3). Risk of death was significantly higher both before and after adjusting for OAC use in patients with any MHC, depression and schizophrenia, whereas there was no difference in the risk of death in patients with anxiety disorder or bipolar disorder, when compared to the matched pairs without MHCs (Figure 1, Table 3). Initiation of OAC therapy was associated with lower risk of IS and death (SHR 0.773 95% CI [0.735–0.813] and HR 0.440 95% CI [0.429–0.452], respectively) among the matched pairs with and without any MHC.

**TABLE 3:** Risk estimates of IS and mortality for MHCs after propensity score matching

Clinical condition	Ischaemic stroke SHR	Mortality HR
Model 1		
No MHC	(Reference)	(Reference)
Any MHC	1.054 (1.005–1.106)	1.210 (1.180–1.242)
Depression	0.970 (0.865–1.086)	1.245 (1.172–1.324)
Bipolar disorder	1.403 (0.951–2.068)	1.085 (0.886–1.327)
Anxiety disorder	0.885 (0.725–1.081)	1.100 (0.987–1.226)
Schizophrenia	0.947 (0.709–1.264)	1.671 (1.465–1.905)
Model 2 (adjusted for OAC)		
No MHC	(Reference)	(Reference)
Any MHC	1.033 (0.985–1.085)	1.149 (1.116–1.175)
Depression	0.961 (0.857–1.077)	1.208 (1.136–1.283)
Bipolar disorder	1.398 (0.947–2.006)	1.068 (0.873–1.308)
Anxiety disorder	0.878 (0.718–1.034)	1.059 (0.950–1.181)
Schizophrenia	0.803 (0.594–1.085)	1.543 (1.352–1.761)

*Note:* Model 1 without adjustments, model 2 adjusted for OAC initiation during follow‐up (before stroke). SHRs estimated with Fine‐Grey subdistribution regression with all‐cause death as competing event, and HRs estimated with Cox proportional hazards regression. 95% confidence intervals in parenthesis.

Abbreviations: HR, hazard ratio; MHC, mental health condition; SHR, subdistribution hazard ratio.

## 4 DISCUSSION

This nationwide cohort study demonstrated that depression, bipolar disorder, anxiety disorder, schizophrenia or a composite of any MHC were not independently associated with the risk of first‐ever IS in patients with incident AF. Depression, schizophrenia and any MHC were associated with higher all‐cause mortality, whereas no association was observed with anxiety and bipolar disorders. The crude incidence of IS was higher in AF patients with any MHC; however, after propensity score matching and adjusting for OAC use the IS risk was comparable with matched comparisons, suggesting that the excess risk derives from higher prevalence of stroke risk factors and comorbidities as well as from lower use OAC therapy.

Previous research on the impact of MHCs on the risk of IS and death in AF patients is limited. Our recently published meta‐analysis reported a 25% higher IS risk in AF patients with any MHC, but these pooled results were based on only three studies with heterogeneity in the used confounding variables and included MHCs.^11^ The study by Schauer et al. reported an increased IS risk in AF patients with psychiatric illness, but their results were based on a small and selected sample of Medicaid recipients from the turn of the millenium.^13^ On the contrary, Søgaard et al. found no significant association between severe depression, bipolar disorder or schizophrenia and IS risk.^12^ However, their study covered only AF and MHC diagnoses from the hospital registries and lacked primary care data as well as the data on patients' socioeconomic status, limiting the generalizability of the results due to possible selection, information and confounding biases. Regarding mortality of patients with AF suffering from MHCs, only one small study by Wändell et al. reported higher all‐cause mortality in depressed men, whereas no difference in mortality was seen in women with depression or in men or women with anxiety disorder.^17^

Therefore, the results of our current study, based on uniquely comprehensive nationwide data from all levels of care substantially, expand our understanding of the impacts of mental illnesses on the risk of IS and death in AF patients. Our principal findings of similar IS risk in AF patients with MHCs are in line with the findings by Søgaard et al. but in contrast with the pooled results of the recent meta‐analysis.^11^, ^12^ Moreover, several studies have associated MHCs with increased IS risk regardless of the presence of AF; however, our study does not confirm these findings in AF patients.^7^, ^8^, ^9^, ^10^ Although previous research on mortality in AF patients with MHCs is limited, our results of higher mortality in patients with depression, schizophrenia and any MHC are in concordance with reports of higher mortality in these patients regardless of the presence of AF.^7^, ^18^, ^19^, ^20^ Especially, schizophrenia has been associated with poor overall survival, corresponding with our observation of more than 50% higher adjusted mortality in AF patients with schizophrenia, when compared to patients without MHC.^19^, ^20^ Our finding of lower crude IS incidence and mortality in AF patients with anxiety disorder is also in line with previous observations on relatively good survival in patients with anxiety disorder without AF.^21^, ^22^

A higher incidence of IS was observed in AF patients with any MHC both before and after propensity score matching. However, after adjusting for OAC use, the risk estimate of IS emerged nonsignificant, indicating that the lower rate of OAC use in patients with any MHC reflects in their high IS rate and that part of these strokes are potentially preventable with adequate OAC coverage.^14^ Correspondingly, in all studied MHC categories, adjusting for OAC use decreased the risk estimates for mortality, suggesting that also part of the higher mortality in these patients may derive from lower utilization of OAC therapy.

The observed higher mortality in AF patients with depression, schizophrenia or any MHC is likely multifactorial. Patients with MHCs have high prevalence of both diagnosed and undiagnosed comorbidities, which are frequently undertreated, and poor treatment compliance often further decreases the benefits of initiated therapies.^23^, ^24^, ^25^, ^26^ Unhealthy lifestyle habits are also common in patients suffering from mental illnesses.^27^, ^28^ Additionally, patients with MHCs have a considerably higher rate of unnatural deaths, including suicides and accidents.^18^ Moreover, some of the interplay between MHCs and adverse outcomes in AF patients may be explained by the ‘heart‐brain‐axis’, which is increasingly acknowledged as an important aspect behind conditions such as Takotsubo and peri‐partum cardiomyopathies.^29^ Future studies are needed to better understand the independent role and mechanisms of different MHCs on risk of death in AF patients.

The main limitations of our study are the challenges inherent to retrospective cohort studies based on administrative data, and thus, our results represent associations and not necessarily causality. Additionally, information bias may be present in the classification of MHC groups using recorded codes; however, we attempted to reduce this bias by using any MHC variable, which also covered patients with purchases of psychotropic medications, although these drugs also have marginal use in other indications. We defined MHCs categorically from medical history, and symptom severity or possible changes in mental health during the study period could not be accounted for. Furthermore, we lacked information on the quality of OAC therapy as well as of other medical and psychological therapies used during follow‐up. Except for diagnosed alcohol abuse disorders, our data lacked information on lifestyle‐related factors. While the national hospital registers in Finland are well‐validated, especially regarding cardiovascular diseases, for the time being the newer primary care register lacks similar validation studies.^30^, ^31^, ^32^ Disparities in the burden of well‐established stroke risk factors between patients with and without MHCs in our study likely affect their risk of IS and death, and although the propensity score matched pairs were similar in several characteristics, residual confounding cannot be excluded. Nonetheless, our data represent a large nationwide cohort comprising all Finnish incident AF patients without history of stroke or TIA from all levels of care, making our results particularly generalizable. Additionally, except for marginal emigration, the used administrative registry data have virtually no loss of follow‐up.

In conclusion, the present nationwide cohort study covering all AF patients in Finland demonstrated that MHCs are not independently associated with higher first‐ever IS risk in patients with incident AF. Additionally, we observed that lower utilization of OAC therapy explains part of the higher crude IS incidence in patients with any MHC. AF patients with any MHC, depression and schizophrenia had higher all‐cause mortality than patients without MHC. Future studies are needed to assess whether interventions aimed at increasing OAC coverage among AF patients with MHCs improve their outcomes.

## AUTHOR CONTRIBUTIONS

Dr Teppo had full access to all the data in the study and takes responsibility for the integrity of the data and the accuracy of the data analysis. Teppo, Jaakkola, Putaala, Mustonen, Haukka, Airaksinen and Lehto involved in concept and design. All authors involved in acquisition, analysis or interpretation of data. Teppo involved in drafting of the manuscript. All authors involved in critical revision of the manuscript for important intellectual content. Teppo, Jaakkola and Biancari involved in statistical analysis. Lehto obtained funding. Jaakkola, Halminen and Haukka involved in administrative, technical or material support. Jaakkola, Putaala, Mustonen, Haukka, Airaksinen and Lehto involved in supervision.

## CONFLICT OF INTEREST

Jukka Putaala: Dr. Putaala reports personal fees from Boehringer Ingelheim, personal fees and other from Bayer, grants and personal fees from BMS‐Pfizer, personal fees from Portola, other from Amgen, personal fees from Herantis Pharma, personal fees from Terve Media, other from Vital Signum, personal fees from Abbott, outside the submitted work. Pirjo Mustonen: Consultant: Roche, BMS‐Pfizer‐alliance, Novartis Finland, Boehringer Ingelheim, MSD Finland. Jari Haukka: Consultant: Research Janssen R&D; Speaker: Bayer Finland. Miika Linna: Speaker: BMS‐Pfizer‐alliance, Bayer, Boehringer Ingelheim. Juha Hartikainen: Research grants: The Finnish Foundation for Cardiovascular Research, EU Horizon 2020, EU FP7. Advisory Board Member: BMS‐Pfizer‐alliance, Novo Nordisk, Amgen. Speaker: Cardiome, Bayer. K.E. Juhani Airaksinen: Research grants: The Finnish Foundation for Cardiovascular Research; Speaker: Bayer, Pfizer and Boehringer Ingelheim. Member in the advisory boards: Bayer, Pfizer and AstraZeneca. Mika Lehto: Consultant: BMS‐Pfizer‐alliance, Bayer, Boehringer Ingelheim, and MSD; Speaker: BMS‐Pfizer‐alliance, Bayer, Boehringer Ingelheim, MSD, Terve Media and Orion Pharma. Research grants: Aarne Koskelo Foundation, The Finnish Foundation for Cardiovascular Research, and Helsinki and Uusimaa Hospital District research fund, Boehringer Ingelheim. Aapo Aro: Research grants: Finnish Foundation for Cardiovascular Research; Speaker: Abbott, Johnson&Johnson, Sanofi, Bayer, Boehringer Ingelheim. Other authors: none.

## Supporting information


Appendix S1

## Data Availability

Because of the sensitive nature of the data collected for this study, requests to access the dataset from qualified researchers trained in human subject confidentiality protocols may be sent to the Finnish national register holders (KELA, Finnish Institute for Health and Welfare, Population Register Center and Tax Register).
