# Supplementary material for: Mental health conditions and risk of first‐ever ischaemic stroke and death in patients with incident atrial fibrillation: A nationwide cohort study
Source: Eur J Clin Invest. 2022 May 7;52(9):e13801. doi: 10.1111/eci.13801 (PMC9539593; doi:10.1111/eci.13801)

**Supplementary material**

**Supplementary Table 1.** Definitions of the comorbidities

**Supplementary Table 2.** Descriptive characteristics of the propensity score matched pairs of patients with and without any MHC

**Supplementary Table 3.** Descriptive characteristics of the propensity score matched pairs of patients with and without depression, bipolar disorder, anxiety disorder and schizophrenia.

**Supplementary Figure 1.** Flow-chart of the patient selection process

**Supplementary Table 1**. Definitions of the comorbidities

|  | ICD-10 | ICPC-2 | Reimbursement code | ATC code |
| --- | --- | --- | --- | --- |
| Hypertension | I10-I15 | K85, K86, K87 | 205 | C03A, C03B, C03DB, C03EA,  C07A, C08CA,  C08D, C09 |
| Dyslipidemia | E78 | T93 | 206 | C10 |
| History of heart failure | I50, I11.0, I13.0, I13.2 | K77 | 201 |  |
| Diabetes | E10-E14 | T89, T90 | 103, 215 | A10 |
| Previous stroke | I63, I64, I69.3-I69.8 | K90 |  |  |
| Vascular disease | I20-I25, I65-I66, I67.2, I70 | K74, K75, K76, K91, K92 | 206 |  |
| Bleeding history | D50.0, D62, D68.3, I60-I62, I69.0-I69.2, I85.0, I86.4, J94.2, K22.1, K22.3, K22.6, K25.0, K25.2,  K25.4, K25.6, K26.0, K26.2, K26.4, K26.6, K27.0, K27.2, K27.4, K27.6, K28.0, K28.2, K28.4, K28.6, K29.0, K62.5, K63.1, K63.3, K92.0-K92.2, N02, R04, R31, R58, S06.2-S06.6, S06.8 |  |  |  |
| Alcohol abuse | F10 |  |  |  |
| Renal failure or dialysis | N18, Z49 |  |  |  |
| Liver cirrhosis or failure | K70.2-K70.4, K71.7, K71.8, K72, K74 |  |  |  |
| Dementia | F00-F03, G30 |  |  |  |

Abbreviations: ATC, anatomic therapeutic chemical; ICD-10, International Classification of Diseases, Tenth Revision; ICPC-2, International Classification of Primary Care, Second Edition.

**Supplementary Table 2.** Descriptive characteristics of patients with and without any MHC before and after propensity score matching

|  | Unmatched cohorts | | | Propensity score matched cohorts | | |
| --- | --- | --- | --- | --- | --- | --- |
| Clinical variables | No MHC  N=164212 | MHCs  N=38942 | Standardized differences | No MHC  N=38552 | MHCs  N=38552 | Standardized differences |
| Age, years | 71.8±13.3 | 71.7±14.6 | 0.007 | 72.4±13.4 | 71.8±14.6 | 0.043 |
| Female gender | 87678 (53.4) | 15980 (41.0) | 0.249 | 15469 840.1) | 15962 (41.4) | 0.026 |
| Chronic kidney disease | 3046 (1.9) | 906 (2.3) | 0.032 | 762 (2.0) | 888 (2.3) | 0.022 |
| Liver disease | 789 (0.5) | 321 (0.8) | 0.042 | 264 (0.7) | 294 (0.8) | 0.009 |
| Alcohol abuse | 4344 (2.6) | 3724 (9.6) | 0.292 | 3370 (8.7) | 3334 (8.6) | 0.003 |
| Hypertension | 124677 (75.9) | 31097 (79.9) | 0.094 | 30922 (80.2) | 30743 (79.7) | 0.011 |
| Dyslipidemia | 73535 (44.8) | 17918 (46.0) | 0.024 | 18006 (46.7) | 17735 (46.0) | 0.014 |
| Diabetes | 33314 (20.3) | 9322 (23.9) | 0.086 | 8871 (23.0) | 9148 (23.7) | 0.016 |
| Heart failure | 27113 (16.5) | 7780 (20.0) | 0.089 | 7464 (19.4) | 7669 (19.9) | 0.013 |
| Vascular disease | 39071 (23.8) | 10111 (26.0) | 0.015 | 9878 (25.6) | 10009 (26.0) | 0.007 |
| HAS-BLED score | 1.7±0.8 | 1.8±0.8 | 0.158 | 1.8±0.8 | 1.8±0.9 | 0.006 |
| CHA_2_DS_2_-VASc score | 3.0±1.6 | 3.3±1.6 | 0.154 | 3.3±1.6 | 3.3±1.6 | 0.022 |
| Income quintiles |  |  | 0.249 |  |  | 0.023 |
| 1 | 31183 (19.0) | 9852 (25.3) |  | 9948 (25.8) | 9660 (25.1) |  |
| 2 | 30892 (18.8) | 8710 (22.4) |  | 8671 (22.5) | 8578 (22.3) |  |
| 3 | 32047 (19.5) | 7905 (20.3) |  | 7632 (19.8) | 7849 (20.4) |  |
| 4 | 34410 (21.0) | 6979 (17.9) |  | 6985 (18.1) | 6970 (18.1) |  |
| 5 | 35680 (21.7) | 5496 (14.1) |  | 5316 (13.8) | 5495 (14.3) |  |
| Cohort entry year | 2003±3 | 2003±3 | 0.015 | 2003±3 | 2003±3 | 0.014 |
| OAC therapy during follow-up | 118111 (72.1) | 24781 (63.8) | 0.178 | 271978 (70.5) | 246582 (64.0) | 0.141 |

Abbreviations: CHA_2_DS_2_-VASc, congestive heart failure, hypertension, age ≥75 years, diabetes, history of stroke or TIA, vascular disease, age 65-74 years, sex category (female): modified HAS-BLED score, hypertension, abnormal renal or liver function, prior stroke, bleeding history, age >65 years, alcohol abuse (no labile INR or concomitant antiplatelet/NSAIDs use, max score 7); MHC, mental health condition; OAC, oral anticoagulant therapy.

**Supplementary Table 3.** Descriptive characteristics of the propensity score matched pairs of patients with and without depression, bipolar disorder, anxiety disorder and schizophrenia.

| Clinical variables | No depression  N=8913 | Depression  N=8913 | Standardized differences | No schizophrenia  N=1329 | Schizophrenia  N=1329 | Standardized differences | No bipolar disorder  N=932 | Bipolar disorder  N=932 | Standardized differences | No anxiety disorder N=3651 | Anxiety disorder  N=3651 | Standardized differences |
| --- | --- | --- | --- | --- | --- | --- | --- | --- | --- | --- | --- | --- |
| Age, years | 69.2±14.4 | 68.8±14.6 | 0.027 | 68.5±15.2 | 68.8±11.7 | 0.024 | 62.7±14.8 | 62.8±12.8 | 0.012 | 65.5±16.4 | 65.0±16.4 | 0.032 |
| Female gender | 5299 (59.4) | 5204 (58.4) | 0.022 | 616 (46.4) | 636 (47.9) | 0.030 | 546 (58.6) | 521 (55.9) | 0.054 | 2223 (60.9) | 2194 (60.1) | 0.017 |
| Chronic kidney disease | 208 (2.3) | 241 (2.7) | 0.024 | 24 (1.8) | 28 (2.1) | 0.022 | 22 (2.4) | 22 (2.4) | 0.000 | 69 (1.9) | 90 (2.5) | 0.039 |
| Liver disease | 109 (1.2) | 110 (1.2) | 0.001 | 11 (0.8) | 10 (0.8) | 0.009 | 10 (1.1) | 11 (1.2) | 0.010 | 35 (1.0) | 39 (1.1) | 0.011 |
| Alcohol abuse | 1553 (17.4) | 1537 (17.2) | 0.005 | 126 (9.5) | 129 (9.7) | 0.008 | 268 (28.8) | 270 (29.0) | 0.023 | 646 (17.7) | 614 (16.8) | 0.023 |
| Hypertension | 7147 (80.1) | 7183 (80.5) | 0.010 | 873 (65.7) | 890 (67.0) | 0.027 | 715 (76.7) | 724 (77.7) | 0.023 | 2946 (80.7) | 2927 (80.2) | 0.013 |
| Dyslipidemia | 4189 (47.0) | 4243 (47.6) | 0.012 | 431 (32.4) | 464 (34.9) | 0.052 | 426 (45.7) | 439 (47.1) | 0.028 | 1615 (44.2) | 1613 (44.2) | 0.001 |
| Diabetes | 2295 (25.7) | 2355 (26.4) | 0.015 | 435 (32.7) | 466 (35.1) | 0.049 | 287 (30.8) | 292 (31.3) | 0.012 | 732 (20.0) | 799 (21.9) | 0.045 |
| Heart failure | 1546 (17.3) | 1622 (18.2) | 0.022 | 396 (29.8) | 412 (31.0) | 0.026 | 155 (16.6) | 153 (16.4) | 0.006 | 536 (14.7) | 561 (15.4) | 0.019 |
| Vascular disease | 2147 (24.1) | 2236 (25.1) | 0.023 | 221 (16.6) | 265 (19.9) | 0.086 | 167 (17.9) | 178 (19.1) | 0.030 | 789 (21.6) | 800 (21.9) | 0.007 |
| HAS-BLED score | 1.8±0.9 | 1.8±0.9 | 0.009 | 1.6±0.9 | 1.6±0.9 | 0.009 | 1.7±1.0 | 1.7±0.9 | 0.003 | 1.7±0.9 | 1.7±0.9 | 0.007 |
| CHA_2_DS_2_-VASc score | 3.1±1.7 | 3.1±1.7 | 0.018 | 3.1±1.7 | 3.1±1.6 | 0.006 | 2.5±1.6 | 2.5±1.5 | 0.003 | 2.9±1.6 | 2.9±1.7 | 0.011 |
| Income quintiles |  |  | 0.044 |  |  | 0.088 |  |  | 0.087 |  |  | 0.053 |
| 1 | 1920 (21.5) | 1836 (20.6) |  | 718 (54.0) | 712 (53.6) |  | 189 (20.3) | 186 (20.0) |  | 736 (20.2) | 697 (19.1) |  |
| 2 | 1999 (22.4) | 2058 (23.1) |  | 328 (24.7) | 350 (26.3) |  | 217 (23.3) | 224 (24.0) |  | 831 (22.8) | 857 (23.5) |  |
| 3 | 1879 (21.1) | 2002 (22.4) |  | 165 (12.4) | 162 (12.2) |  | 191 (20.5) | 194 (20.8) |  | 755 (20.7) | 817 (22.4) |  |
| 4 | 1897 (21.3) | 1816 (20.4) |  | 83 (6.2) | 61 (4.6) |  | 212 (22.7) | 185 (19.8) |  | 794 (21.7) | 779 (21.3) |  |
| 5 | 1223 (13.7) | 1206 (13.5) |  | 35 (2.6) | 44 (3.3) |  | 123 (13.2) | 143 (15.3) |  | 535 (14.7) | 501 (13.7) |  |
| Cohort entry year | 2014±3 | 2014±3 | 0.032 | 2013±3 | 2014±3 | 0.013 | 2014±3 | 2014±3 | 0.038 | 2014±3 | 2014±3 | 0.022 |
| OAC therapy during follow-up | 6099 (68.4) | 5748 (64.5) | 0.083 | 925 (69.6) | 791 (59.5) | 0.212 | 603 (64.7) | 596 (63.9) | 0.017 | 2381 65.2) | 2198 (60.2) | 0.104 |
| Abbreviations: CHA_2_DS_2_-VASc, congestive heart failure, hypertension, age ≥75 years, diabetes, history of stroke or TIA, vascular disease, age 65-74 years, sex category (female): modified HAS-BLED score, hypertension, abnormal renal or liver function, prior stroke, bleeding history, age >65 years, alcohol abuse (no labile INR or concomitant antiplatelet/NSAIDs use, max score 7); MHC, mental health condition; OAC, oral anticoagulant therapy. | | | | | | | | | | | | |

**Supplementary Figure 1.** Flow-chart of the patient selection process


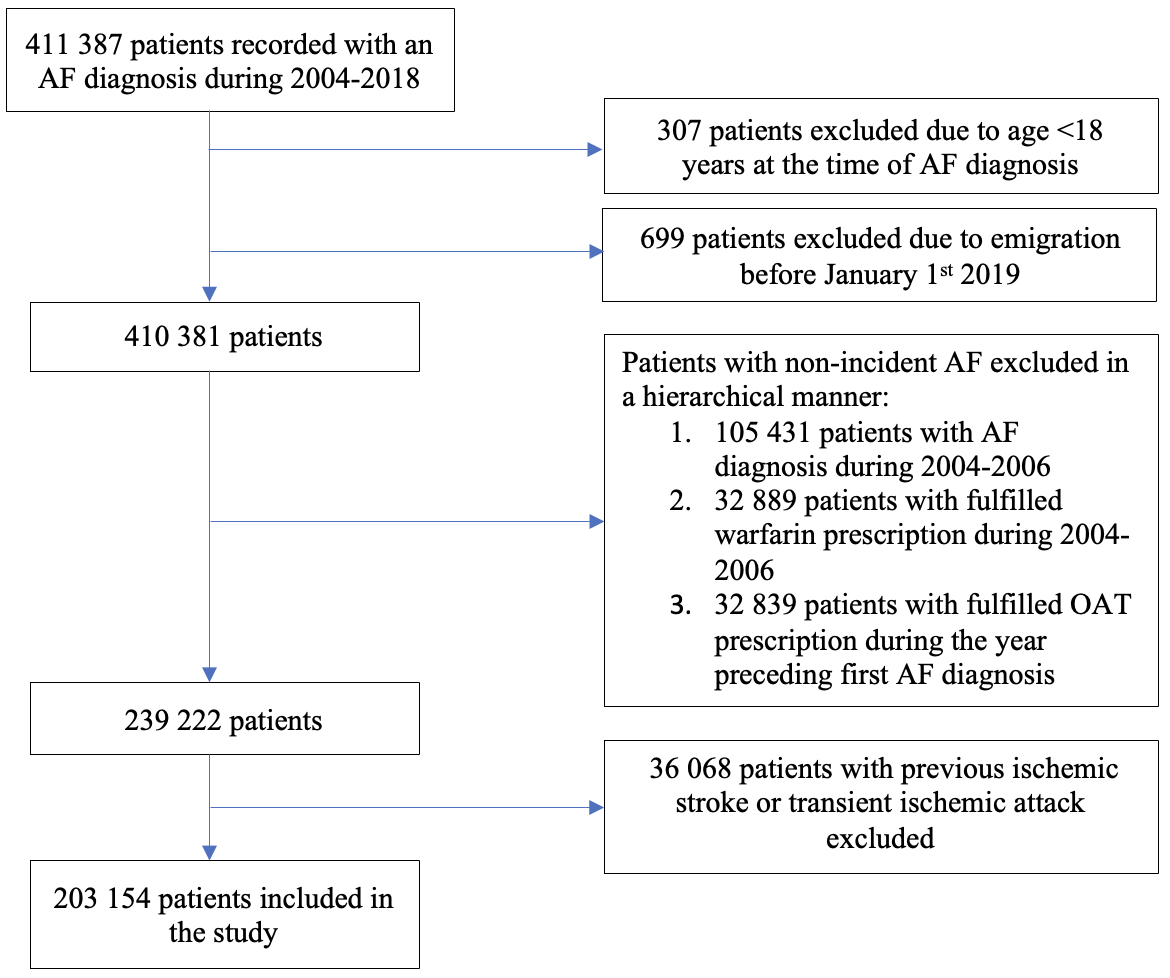

Supplement: Supplementary file 1 — Appendix S1 [file ECI-52-e13801-s001.docx]
